# Tooth Extraction in Patients on Oral Anticoagulants: Prospective Study Conducted in 108 Brazilian Patients

**DOI:** 10.5402/2011/203619

**Published:** 2011-05-29

**Authors:** Claudio Maranhão Pereira, Patrícia Freire Gasparetto, Danilo Santos Carneiro, Maria Elvira P. Corrêa, Cármino Antônio Souza

**Affiliations:** ^1^School of Dentistry, Paulista State University, Goiânia, GO, Brazil; ^2^Estácio de Sá University, Goiânia, GO, Brazil; ^3^Hematology and Hemotherapy Center, State University of Campinas, Campinas, SP, Brazil

## Abstract

*Introduction*. Dental treatment performed in patients receiving continuous oral anticoagulant drug therapy is becoming increasingly common in dental offices. For these patients it is imperative to carry out careful anamnesis, as well as a multiprofessional clinical evaluation with regard to the risk and control of hemorrhagic or thromboembolic episodes. *Objectives and Material and Methods*. The aim is to evaluate postextraction hemorrhagic or thromboembolic episodes in patients who have been on anticoagulant medications for an uninterrupted period of 48 months. *Results*. Among the 108 patients evaluated, 215 extractions were performed in which there was only one case of postoperative bleeding. Warfarin was used by 98 patients; Warfarin associated with salicylic acetic acid by 9 patients and salicylic acetic acid in only 1 patient. As regards the serologic tests performed, International Normalized Ratio (INR) ranged from 0.8 to 4.9, with a mean of 3.15. *Conclusion*. Extractions in patients on oral anticoagulants must be performed in the least traumatic manner possible. It is not necessary to stop anticoagulant therapy to perform extractions. Local hemostasis techniques, such as obliterative sutures alone are sufficient to prevent hemorrhagic complications.

## 1. Introduction

Ambulatory dental surgery in patients with hemostatic alterations, and who use oral anticoagulant drugs (OAC) has become a constant practice over the last few years, demanding a specific approach by the dentist and interdisciplinary interaction with the various health teams that follow up the patient [1]. 

There is discussion on how to perform dental treatment safely in patients on anticoagulants. Some time ago, the American College of Cardiology (ACC) and the American Heart Association (AHA) developed a set of practical guidelines that propose to develop, review, and update protocols for cardiovascular diseases and assist with clinical procedures. Some protocols suggest stopping use of the drug, in addition to the administration of vitamin K or heparin before procedures with potential for hemorrhage. However, these alterations may increase the chance of an episode of thromboembolism in patients [1, 2]. 

None of these schemes is risk-free, which makes it imperative to carry out a complete evaluation of the patient's systemic condition, followup of his/her degree of anticoagulation, and classification of the amplitude of surgical trauma involved in the dental procedure to be performed. Protocols have been researched to guarantee a treatment that prevents the occurrence of hemorrhages, and at the same time, not expose the patient to the risk of thromboembolism. Interrupting anticoagulant therapy, thereby exposing the patient to an unnecessary risk of thromboembolism, is not a cautious attitude. Many authors have demonstrated that it is safe to perform the majority of dental surgical procedures without risk of severe hemorrhages when the International Normalized Index (INR) is within the therapeutic levels [3].

Patients who use OAC have their therapy monitored by measuring the Prothrombin Time (PT). This test measures the time for clot formation from VII factor activation to fibrin coagulum formation. Due to variations in the methodology, reagents, and instruments used in each laboratory, a normalization ratio was established for PT measurements (INR) [4]. There is increased risk of thromboembolic events when the INR is below the therapeutic level; when it is above, the risk of hemorrhages increases dramatically, particularly in the elderly [5, 6].

Based on the facts set forth, we proposed to evaluate the quantity and severity of hemorrhagic episodes after tooth extraction in patients on anticoagulant medications and who have been under medical and dental treatment at the Hemotherapy and Hematology Center (HEMOCENTRO) of the State University of Campinas, SP, Brazil (UNICAMP) for an uninterrupted period of 48 months. 

## 2. Material and Methods

An evaluation was made of 108 patients undergoing treatment with anticoagulant drugs, who have been receiving medical attendance at the Hemotherapy and Hematology Center of the State University of Campinas, SP, for a continuous period of 48 months. 

All the institutionalized patients, by local protocol, receive dental evaluation and treatment. In all, among the 108 patients attended by the dental service during this period, 215 extractions were performed, and all the procedures were carried out without stopping the anticoagulant drug. 

During the first consultation, routine oral exams were performed, such as PI (plaque index), GI (gingival index), DMF-T (decayed, missing and filled teeth) and panoramic radiographs [7, 8]. Before extraction, the INR was requested and the procedure was only performed if it were within the acceptable limit for each patient. Because the patients who were on oral anticoagulant drugs had prosthetic valves, prophylactic antibiotics were administered, in accordance with the AHS (American Hearth Society); that is to say, a dose equivalent to 2 g one hour before the surgical procedure [9]. The extractions were performed with regional block anesthetic techniques and, if necessary, infiltrative intraligamentary anesthesia. The surgical technique was performed in the least traumatic manner possible. After removal of the tooth, obliterative suturing (multiples and several sutures in the one region) was performed and the patient was recommended to use an ice pack on the face, physical rest, and to eat cold food of a soft consistency. No other local hemostatic was used, except for the suture itself.

All the patients were informed of the nonmandatory nature of the research. It was also emphasized that noncooperation with the research would not result in any changes in their medical clinical treatment. The research was submitted to the Research Ethics Committee of the Medical Science Faculty of the State University of Campinas for approval, which was granted.

## 3. Results

Of the 108 patients, 61 were women and 47 men. The patients' ages ranged from 13 to 80 years (median of 48.5 years). The most frequent base systemic disease was thrombosis, followed by valvulopathy, cerebrovascular accident (CVA), cardiopathy, Chagas disease, and pulmonary embolism (Table 1). Warfarin was used by 98 patients; Warfarin was associated with salicylic acetic acid by 9 patients and salicylic acetic acid in only 1 patient. 

As regards the oral condition of these patients, the mean DMF-T was 13.8. Evaluation of the plaque and gingival indexes is shown in Tables 2 and 3. With regard to the serological tests performed, the INR ranged from 0.8 to 4.9, with a mean of 3.15 (Table 4). Of the 215 extractions there was postoperative bleeding in only 1 case. This patient was a 72-year-old man, the IRN was 1.57 and the tooth 37 was the one extracted. 

## 4. Discussion

In patients who make continuous use of oral anticoagulant drugs, it is imperative to carry out careful anamnesis, as well as a multiprofessional clinical evaluation with regard to the risk and control of hemorrhagic or thromboembolic episodes [10].

It is imperative to have the patient's degree of anticoagulation under medical control, and it must be checked periodically to verify whether the necessary hemostatic therapeutic level is being maintained. For this purpose, the prothrombin time is used, the result of which may be expressed in seconds, in prothrombin activity or in INR, which must be performed in a maximum interval of 4 weeks, as recommended by the American College of Chest Physicians [3]. Generally speaking, the therapeutic interval of INR should remain between 2.0 and 3.5, but depending on the type of disease presented by the patient, higher INR values are considered therapeutic [5–7]. 

Lippert and Gutschik published recommendations in which the INR should not be higher than 4.0, and preferably lower than 3.0, before the patient on an anticoagulant is submitted to dental procedures with high risk of bleeding [11]. 

The recommendations of some authors for various dental surgical procedures indicate that for simple extractions, or when minimal bleeding is expected, an INR lower than 4.0 is acceptable. For cases of moderate bleeding, included and impacted third molar surgeries or multiple extractions, the INR should be reduced; in cases where greater hemorrhage is expected, an INR lower than 3.0 is indicated; and when the INR is over 5.0, no surgeries should be performed [5, 6, 11–13]. 

When evaluating the IRN results of our patients, these values ranged from 0.8 to 4.9, with a mean of 3.15. We observed that in the specialized literature there is no unanimity as regards the maximum values of IRN considered safe for the patient to be submitted to tooth extractions. Nevertheless, even in those patients with IRN over 4.0, which would be considered high by some authors [6, 11], we had no episodes of hemorrhages. On the other hand, the only patient who presented hemorrhage had an IRN of 1.57, a value considered safe by all the researched authors [5, 6, 11–13]. 

Elderly patients are among those who most benefit from anticoagulant treatment; however, they are also among those with the greatest risk of hemorrhagic complications [6, 14]. Apparently we had no explicit factor that would justify the hemorrhagic episode in the only case we investigated. We can emphasize that the patient was elderly (72 years of age) which corroborates the literature stating that they are the patients most susceptible to hemorrhages.

Many authors are sufficiently concerned to point out the necessity of using an atraumatic surgical technique and the application of local conventional measures to control hemostasis, in which the adequate suture is extremely important [1, 14–17].

Maintenance of the anticoagulant medication has been recommended by an increasing number of researches, which observed a minimal incidence of hemorrhagic episodes after surgeries, in which the patients' values in PT and/or in INR were within the therapeutic indexes [10, 14, 16]. This protocol has been even further reinforced by authors who emphasize the use of local hemostatics, affirming their efficiency in the prevention and control of postoperative hemorrhages [17–19]. Our survey showed similar results with a minimum rate of postoperative bleeding (one case) controlled by local measures with plugging. These guidelines are also recommended by the British Committee for Standards in Hematology, which states the risk of bleeding in these patients when maintaining the INR between 2 and 4 is low for dental surgeries, and that the interruption of anticoagulant therapy would not be justified due to the increase in the risk of thrombosis.

It is imperative to evaluate the risks of transoperative and postoperative hemorrhage, as well as the amount of surgical trauma to which this patient would be subjected in order to establish an adequate attendance protocol. Therefore, we point out that performing a surgical procedure with the least amount of trauma possible, strict observance of all the steps of the surgical procedure, including adequate suturing, and the patient's compliance with the postoperative recommendations must be primordial factors to consider at all times in all patients, and especially in those on anticoagulant medications.

## 5. Conclusion

Extractions in patients on oral anticoagulants must be performed in the least traumatic manner possible. It is not necessary to stop anticoagulant therapy to perform extractions. Local hemostatic techniques such as obliterative sutures alone are sufficient.

## Figures and Tables

**Table 1:** List of base systemic diseases in patients who were submitted to tooth extractions.

Base disease	Number of patients
Thrombosis	34
Valvulopathy	30
CVA	30
Cardiopathy	8
Cirrhosis	3
Chagas Disease	2
Pulmonary embolism	1

Total	108

**Table 2:** Plaque index of patients who underwent tooth extractions.

Plaque index	Number of patients
0	16
1	46
2	29
3	17

Total	108

**Table 3:** Gingival index of patients who underwent tooth extractions.

Gingival index	Number of patients
0	23
1	55
2	16
3	14

Total	108

**Table 4:** Correlation between INR of patients with the number of tooth extractions performed.

INR	Number of tooth extractions
0.5–1.0	7
1.01–1.5	36
1.51–2.0	50
2.01–2.5	58
2.51–3.0	34
3.01–3.5	17
3.51–4.0	9
4.01–4.5	2
4.51–5.0	2

Total	215
